# Crystal Structure of Human EOLA1 Implies Its Possibility of RNA Binding

**DOI:** 10.3390/molecules24193529

**Published:** 2019-09-29

**Authors:** Minju Kim, Sang Ho Park, Joon Sung Park, Hyun-Jung Kim, Byung Woo Han

**Affiliations:** 1 Research Institute of Pharmaceutical Sciences, College of Pharmacy, Seoul National University, 1 Gwanak-ro, Gwanak-gu, Seoul 08826, Korea; mkim13@snu.ac.kr (M.K.); sanghop@med.umich.edu (S.H.P.); wingpjs@snu.ac.kr (J.S.P.); 2 Department of Biological Chemistry, University of Michigan, Ann Arbor, MI 48109, USA; 3 College of Pharmacy, Chung-Ang University, 84 Heukseok-ro, Dongjak-gu, Seoul 06974, Korea; hyunjungkim@cau.ac.kr

**Keywords:** activating signal cointegrator-1 homology (ASCH) domain, endothelial-overexpressed lipopolysaccharide-associated factor 1 (EOLA1), lipopolysaccharide (LPS), RNA-binding domain

## Abstract

Human endothelial-overexpressed lipopolysaccharide-associated factor 1 (EOLA1) has been suggested to regulate inflammatory responses in endothelial cells by controlling expression of proteins, interleukin-6 and vascular cell adhesion molecule-1, and by preventing apoptosis. To elucidate the structural basis of the EOLA1 function, we determined its crystal structure at 1.71 Å resolution and found that EOLA1 is structurally similar to an activating signal cointegrator-1 homology (ASCH) domain with a characteristic β-barrel fold surrounded by α-helices. Despite its low sequence identity with other ASCH domains, EOLA1 retains a conserved ‘**G**x**K**xx**E**x**R**’ motif in its cavity structure. The cavity harbors aromatic and polar residues, which are speculated to accommodate nucleotide molecules as do YT521-B homology (YTH) proteins. Additionally, EOLA1 exhibits a positively charged cleft, similar to those observed in YTH proteins and the ASCH protein from *Zymomonas* *mobilis* that exerts ribonuclease activity. This implies that the positively charged cleft in EOLA1 could stabilize the binding of RNA molecules. Taken together, we suggest that EOLA1 controls protein expression through RNA binding to play protective roles against endothelial cell injuries resulting from lipopolysaccharide (LPS)-induced inflammation responses.

## 1. Introduction

Endothelial-overexpressed lipopolysaccharide-associated factor 1 (EOLA1) is overexpressed in human umbilical vein endothelial cells (HUVEC) stimulated with lipopolysaccharide (LPS) [1]. LPS is found in the outer membrane of Gram-negative bacteria and mediates apoptosis in endothelial cells [2], which is implicated in endothelial injuries, sepsis, shock, and multiple organ dysfunction [3,4]. LPS could also induce expression of specific adhesion molecules and inflammatory cytokines such as interleukin (IL)-6, vascular cell adhesion molecule-1 (VCAM-1), and monocyte chemotactic protein (MCP)-1 in endothelial cells, which results in fibrosis, apoptosis of the endothelial cells, and recruitment of immune cells [5,6,7,8,9,10]. Interestingly, overexpressed EOLA1 was reported to inhibit IL-6 expression and apoptosis of LPS-treated HUVEC potentially via the metallothionein-2A (MT-2A)-mediated pathway, which implied that EOLA1 prevents cell death and protects endothelial cells from stresses such as inflammation and cell injuries [10]. MT-2A is a 61-amino acid metal binding protein and has been reported to interact with EOLA1 as deduced from yeast two-hybrid assay and co-immunoprecipitation experiments [1]. However, the mechanism of EOLA1 function still remains unclear.

EOLA1 is a member of the activating signal cointegrator-1 homology (ASCH) domain superfamily [11]. Activating signal cointegrator-1 (ASC-1, a.k.a. thyroid receptor-interacting protein 4, TRIP-4) is a mammalian-conserved transcription coactivator and has been known to interact with nuclear hormone receptors, other coactivators including steroid receptor coactivator-1 and cAMP response element-binding protein (CREB)-binding protein/p300, and transcription factors, possibly resulting in the formation of a supra-coactivator complex [12,13]. ASC-1 contains a unique β-barrel domain at its C-terminus (residues 435–581, PDB ID: 2E50), which exhibits a topological similarity to an RNA-binding pseudouridine synthase and archaeosine transglycosylase (PUA) domain. As a coactivator, ASC-1 was predicted to interact with RNAs through the PUA-like domain [11]. Recently, an ASCH protein from *Zymomonas mobilis* (*Zm*ASCH) was reported to exhibit nuclease activity on single-stranded RNA [14]. In addition, the EVE domain (named after PDB ID: 2EVE of protein PSPTO5229 from *Pseudomonas syringae*) and the splicing factor YT521-B homology (YTH) domain also adopted similar topologies to the PUA domain despite their low amino acid sequence identities. In particular, the YTH domain was shown to be capable of binding RNA, which has been further supported by the determination of its RNA-complexed structures [15,16,17,18].

To shed light on the functions of EOLA1, we first determined the crystal structure of EOLA1 at 1.71 Å resolution using the single-wavelength anomalous dispersion (SAD) method. The structure of EOLA1 shares a core cavity and a positively charged cleft with the ASCH domain and exhibits structural differences in other parts. We compared the EOLA1 structure with other PUA-like proteins, especially those in complex with RNA molecules. Our structural analyses on EOLA1 imply that EOLA1 could be an RNA-binding protein and may play a regulatory role in RNA metabolism to protect vein endothelia that has been invaded by LPS-positive bacteria.

## 2. Results

### 2.1. Endothelial-Overexpressed Lipopolysaccharide-Associated Factor 1 (EOLA1) Contains Typical Activating Signal Cointegrator-1 Homology (ASCH) Structure

EOLA1 (gene, CXorf40A) contains 158 amino acids and has been predicted to possess the ASCH domain, a newly classified domain similar to the PUA domain that plays an RNA-binding role to regulate gene expression [11]. To gain insight on the functions of EOLA1, we determined the crystal structure of EOLA1 at 1.71 Å resolution. EOLA1 crystals belonged to the *P*4_1_2_1_2 space group (unit cell parameters; a = b = 49.77 Å, c =175.71 Å, α = β = γ = 90°). The statistics for data collection and model refinement are summarized in Table 1. 

**Figure 1:**
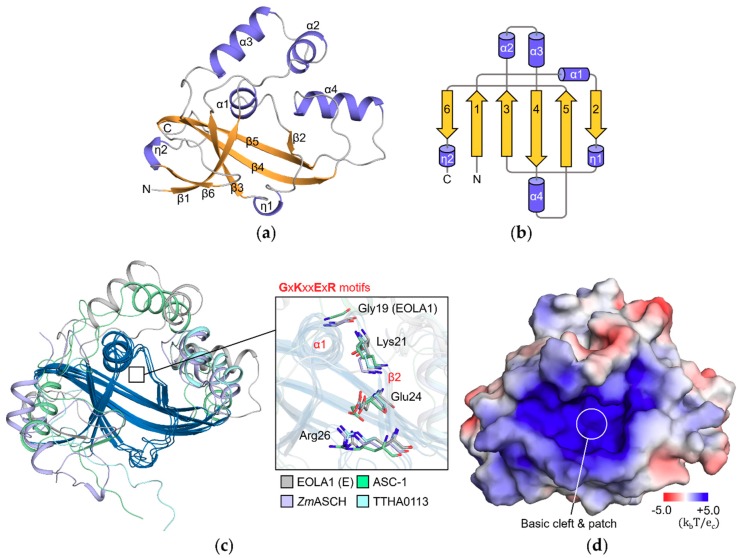
Overall structure of EOLA1. (**a**) Crystal structure of EOLA1 represented in cartoon representation. Four α-helices (α1–α4) and two 3_10_-helices (η1 and η2) are colored in slate, and six β-strands (β1–β6) are colored in bright orange. (**b**) Topology of EOLA1. Helices and strands are colored in slate and bright orange, respectively. (**c**) Superimposition of EOLA1 with structurally similar proteins. The regions shared by EOLA1, activating signal cointegrator-1 (ASC-1), *Zm*ASCH (ASCH protein from *Zymomonas mobilis*), and TTHA0113 are colored in blue, and the other regions of EOLA1, ASC-1, *Zm*ASCH, and TTHA0113 are colored in gray, palegreen, light blue, and pale cyan, respectively. In the right panel, a magnified view of the conserved ‘**G**x**K**xx**E**x**R**’ motifs are shown. The motif residues (Gly, Lys, Glu, and Arg) of EOLA1 (labeled), ASC-1, *Zm*ASCH, and TTHA0113 are shown as gray, palegreen, light blue, and pale cyan stick model, respectively. Nitrogen and oxygen atoms are colored in blue and red, respectively. (**d**) Electrostatic potential surface of EOLA1 calculated using the APBS program. Positive and negative surfaces are colored in blue and red, respectively. The color scales bar is shown below.

EOLA1 comprises four α-helices (α1–α4), two 3_10_-helices (η1 and η2), and six β-strands (β1–β6) that form a β-barrel structure flanked by α-helices (Figure 1a,b). The Dali server analyses [19] showed that ASC-1 (Z-score: 12.4, PDB ID: 2E5O), ASCH protein of *Zymomonas mobilis Zm*ASCH (Z-score: 10.4, PDB ID: 5GUQ), and hypothetical protein TTHA0113 of *Thermus thermophilus* HB8 (Z-score: 9.2, PDB ID: 2DP9) are structurally most similar to EOLA1 among the reported protein structures. When superimposed, they share the characteristic mixed β-sheet core of the ASCH domain containing five strands (β1–β5 in EOLA1), and one α-helix (α1 in EOLA1) located between the first and second β-strands (Figure 1c). Helix η1 of EOLA1 seems to be overlapped with loops of the other superimposed proteins. The structures also contain the conserved ‘**G**x**K**xx**E**x**R**’ sequence of the ASCH domain [11,20] (Figure 1c). This conserved motif is located in the α1-β2 region of the ASCH domain that forms a cavity structure (Figure 1d).

**Figure 2:**
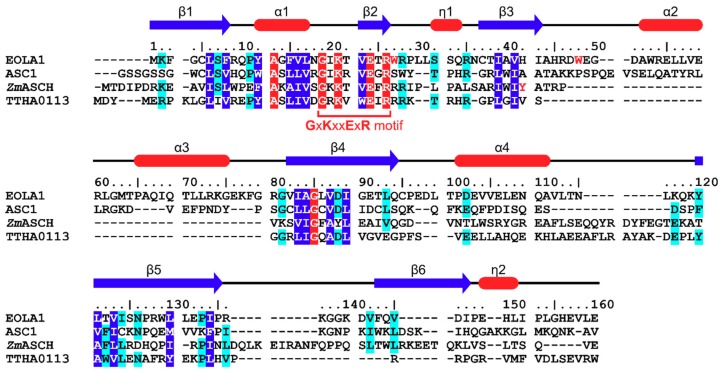
Sequence alignment of EOLA1 with structurally similar proteins. Sequences alignment of EOLA1, ASC-1, *Zm*ASCH, and TTHA0113 based on Dali analyses [19]. The secondary structure of EOLA1 is shown above its sequence; helices and β-strand are represented as red tubes and blue arrows, respectively. Identical and conserved residues among four proteins are marked with red and blue boxes, respectively. Conserved residues among three of four proteins are marked with cyan boxes.

The sequences of EOLA1 and the three structurally similar proteins are aligned based on the Dali analyses (Figure 2). Along with the ‘**G**x**K**xx**E**x**R**’ motif residues, Ala13 and Gly85 are strictly conserved among the four proteins. As these residues are located on the interface between the core β-sheet and helix α1, they may contribute to the conformation of helix α1 and the resultant cavity structure. Additionally, we observed that Leu6, Phe8, Tyr12, Phe15, Val16, Leu17, Val23, Leu39, Val41, Ile83, Ala84, Val87, Ile89, Leu121, Val123, Leu130, and Ile134 of EOLA1 are conserved among the four proteins and are located on the core β-strands or helix α1 (Figure 2). EOLA1 residues conserved among three of the four proteins include Lys2, Ser7, Pro11, Arg28, Ser32, Arg35, Gly81, Asp88, Leu93, Asp102, Tyr120, Ile124, Asn126, Pro133, Val142, and Val145, most of which are also located on or around the conserved cavity. The structural conservation of the cavity in these ASCH domains strongly suggests that this characteristic cavity structure is involved in the function of the ASCH domain.

Interestingly, EOLA1, ASC1, *Zm*ASCH, and TTHA0113 show varying sequences and topologies except in the core cavity: between β3 and β4, between β4 and β5, and at the C-terminus of β5 in EOLA1. This reflects the different subclasses of these four proteins under the ASCH family where EOLA1 is classified under subclass 4 [11].

### 2.2. Structural Analyses Imply That RNA-Binding Mode of EOLA1 is Different from That of Pseudouridine Synthase and Archaeosine Transglycosylase (PUA) Domains

**Figure 3:**
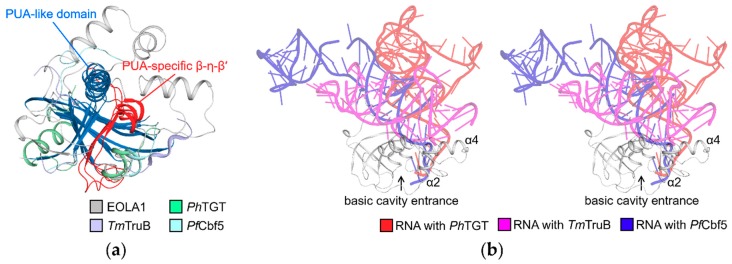
Structural comparison between EOLA1 and pseudouridine synthase and archaeosine transglycosylase (PUA) domain proteins. (**a**) Superimposition of EOLA1 with PUA domains in complex with RNAs. PUA domains of *Pyrococcus horikoshii* archaeosine tRNA-guanine transglycosylase (*Ph*TGT, PDB ID: 1J2B), *Thermotoga maritima* tRNA pseudouridine synthase TruB (*Tm*TruB, PDB ID: 1R3E), and *Pyrococcus furiosus* probable tRNA pseudouridine synthase B (*Pf*Cbf5, PDB ID: 2HVY) are superimposed with EOLA1. Common topology structures shared by the four proteins are labeled as the PUA-like domain and colored in blue. The PUA-specific β-η-β′ that is conserved in PUA domains but not in EOLA1 are colored in red. The other parts of EOLA1, *Ph*TGT, *Tm*TruB, and *Pf*Cbf5 are colored in gray, pale green, light blue, and pale cyan, respectively. (**b**) Stereo-view of EOLA1 superimposed with RNA molecules complexed with PUA domains. RNA molecules complexed with *Ph*TGT, *Tm*TruB, and *Pf*Cbf5 are colored in red, magenta, and blue, respectively. The complexed PUA domains are not shown.

Previous studies showed that ASCH domains are highly similar to PUA domains structurally [11]. This similarity is observed in the five-stranded core β-sheet and the helix between the β1 and β2 strands, which is also observable in the EVE domain and EVE-like YTH domains [20]. Consequently, ASCH domains were predicted to harbor RNA-binding properties like the PUA domain. To examine the possibility of RNA binding in EOLA1, we compared the structures of EOLA1 and PUA domains. As an RNA-recognition motif found in archaea, bacteria, and eukaryotes [21], several PUA domain structures such as *Pyrococcus horikoshii* archaeosine tRNA-guanine transglycosylase (PDB ID: 1J2B), *Thermotoga maritima* tRNA pseudouridine synthase TruB (PDB ID: 1R3E), and *Pyrococcus furiosus* probable tRNA pseudouridine synthase Cbf5 (PDB ID: 2HVY) were determined in complex with tRNA [22,23,24]. Superimposition of EOLA1 onto these three PUA-RNA complexes revealed that PUA domains have a PUA-specific strand that occupies their cavity structure (Figure 3a). Additionally, the interfaces between PUA domains and RNA molecules are overlapped and sterically collide with the non-conserved regions of EOLA1 (Figure 3b). Collectively, it seems that the binding modes of PUA domains are not shared with EOLA1.

### 2.3. Structural Comparison with Zygosaccharomyces Rouxii YTH Domain (ZsYTH) Revealed a Cavity in EOLA1 for A Base Substrate

We next superimposed our EOLA1 structure onto the *Zygosaccharomyces rouxii* YTH domain (*Zs*YTH) structure in complex with methyladenosine RNA (PDB ID: 4U8T), which showed the highest Z-score of 5.8 among available structures of ASCH, EVE, and YTH proteins in complex with an RNA-oligomer from our Dali analyses (Table 2).

**Figure 4:**
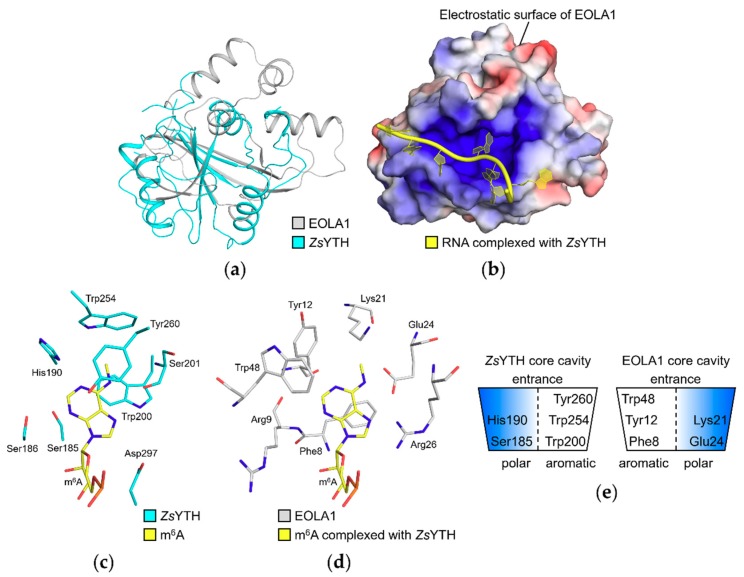
Prediction of interaction of EOLA1 with nucleotides. (**a**) Superimposition of EOLA1 and *Zygosaccharomyces rouxii* YTH domain (*Zs*YTH) structures. The crystal structures of EOLA1 and *Zs*YTH (PDB ID: 4U8T) in complex with RNA are shown as gray and cyan cartoon models, respectively. The RNA molecule is not shown. (**b**) The electrostatic potential surface of EOLA1 superimposed with RNA. Negative and positive surfaces are colored in red and blue, respectively. The RNA molecule complexed with *Zs*YTH is shown as a yellow cartoon, and *Zs*YTH structure is not shown. (**c**) The core-cavity residues of *Zs*YTH. The *Zs*YTH residues forming hydrogen bonds or stacking interactions with the methylated adenosine are shown as cyan sticks. The N6-methylated adenine (m^6^A) is shown as yellow sticks. Nitrogen and oxygen atoms are colored in blue and red, respectively. (**d**) The core-cavity residues of EOLA1. The EOLA1 residues forming stacking or hydrogen-bond interactions with the m^6^A-RNA model from the *Zs*YTH-EOLA1 superimposed structure (**b**) are shown as gray sticks. Nitrogen and oxygen atoms are colored as in (**c**). (**e**) Schematic representation of core cavities of EOLA1 and *Zs*YTH. Aromatic and polar sides in the cavities are colored in white and blue, respectively.

Interestingly, we could observe that the RNA molecule bound to *Zs*YTH is well accommodated when superimposed onto the EOLA1 structure (Figure 4a,b). The methylated adenine, the target nucleotide of *Zs*YTH, extends into the core cavity of EOLA1 while the phosphodiester backbone is located within the basic cleft of EOLA1.

In the recognition mode of RNA by *Zs*YTH, the methylated adenine moiety is accommodated by the cavity containing α1-β2 (numbered based on the domain structure) [15]. In the cavity, three aromatic residues Trp200, Trp254, and Tyr260 in *Zs*YTH form an aromatic cage that captures the base of the methylated adenine. This aromatic cage, together with residues Ser185, Ser186, His190, Ser201, and Asp297, defines the binding pocket for the methylated adenine (Figure 4c). In the EOLA1 structure, we observed that Phe8, Tyr12, and Trp48 in the cavity structure could form an alternative aromatic cage to that of ZsYTH (Figure 4d). Furthermore, polar residues Arg9, Glu24, and Arg26 of EOLA1 could play similar roles of ZsYTH residues, such as Ser186, Ser201, Ser202, and Asp297, forming hydrogen bonds with RNA. Notably, Glu24 and Arg26 of EOLA1 are from the ASCH-specific ‘**G**x**K**xx**E**x**R**’ motif.

We hypothesize that aromatic and hydrogen-bond forming residues in ZsYTH that interact with the methylated adenine could be substituted with similarly positioned interaction pairs/residues from opposite sides of the cavity structure in EOLA1 (Figure 4c–e). In detail, the Ser185 (left-hand side)-Trp200 (right-hand side) pair of ZsYTH could be substituted with the Phe8 (left-hand side)-Glu24 (right-hand side) pair of EOLA1. The His190 (left-hand side, a hydrogen-bond forming residue with RNA in ZsYTH)-Trp254 (right-handed side) pair of ZsYTH could be substituted with the Tyr12 (left-hand side)-Lys21 (right-hand side) pair of EOLA1. Lastly, Trp48 of EOLA1 (located in the left-hand side of its cavity structure) could be a substitute interacting residue for Tyr260 of ZsYTH (located in the right-hand side of its cavity structure). Our structural analysis collectively implies that EOLA1 could recognize substrates slightly similar to those of ZsYTH, but with an opposite arrangement of interacting aromatic and polar residues in its substrate-binding cavity. Additionally, conservation of Phe8 and Lys21 of EOLA1 among the ASCH domain subfamily 4 implies the importance of these two residues in our probable substrate-recognition mode of EOLA1 [11].

### 2.4. Basic Patch of EOLA1 Could Provide a Binding Interface for Nucleic Acid Backbones

**Figure 5:**
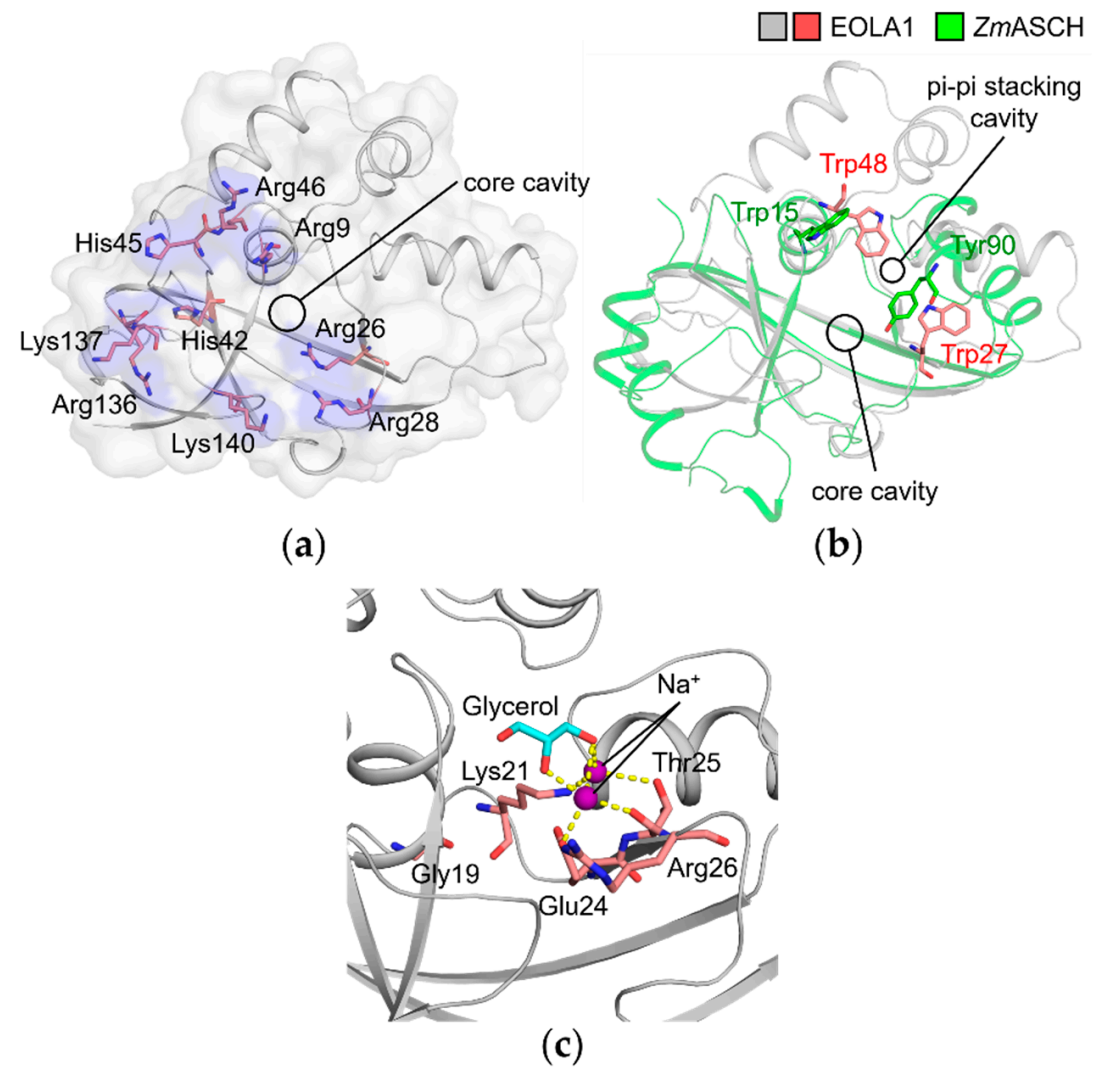
Structural implications of binding between EOLA1 and RNA molecules. (**a**) The basic residues surrounding the core cavity of EOLA1. The residues are shown as salmon sticks and their exposing surfaces are colored in blue. Nitrogen and oxygen atoms are colored in blue and red, respectively. (**b**) The probable pi-pi stacking crevices of EOLA1 and *Zm*ASCH. Superimposed structures of EOLA1 and *Zm*ASCH are shown in gray and green cartoons, respectively. Trp27 and Trp48 of EOLA1 are shown as salmon sticks, and Trp15 and Tyr90 of *Zm*ASCH are shown as green sticks. Nitrogen and oxygen atoms are colored as in (**a**). (**c**) Ligands at the core cavity of EOLA1 in the crystal structure. Gly19, Lys21, Glue24, Thr25, and Arg26 in the ‘**G**x**K**xx**E**x**R**’ motif of EOLA1 are represented as salmon sticks with gray cartoon representation of EOLA1. The glycerol and two sodium ions are colored in cyan and purple, respectively. Nitrogen and oxygen atoms are colored as in (**a**).

The possibility of nucleic acid binding to EOLA1 can be supported by the basic cleft and patch that could provide an electrostatic interacting surface for phosphodiester backbones of nucleic acid as observed in *Zs*YTH [15] (Figure 4b). A similar basic patch is observable in *Zm*ASCH, which exhibits nuclease activities on single-stranded RNA. [14]. Arg9, Arg26, Arg28, His42, His45, Arg46, Arg136, Lys137, and Lys140 of EOLA1 protrude out towards the protein surface and form a basic patch that surrounds the core cavity (Figure 5a).

A structural comparison of EOLA1 with *Zm*ASCH reveals a pi-pi interaction site in EOLA1 that is suspected to accommodate an adjacent base at the entrance of the core cavity. The binding mode between *Zm*ASCH and an RNA heptamer was previously predicted based on molecular dynamics simulation [14]. In the prediction, a hydrophobic pocket formed by Trp15 and Tyr90 in *Zm*ASCH accommodates an adenine base, resulting in the Trp15-adenine-Tyr90 stacking interaction. These two residues are not conserved in EOLA1 when the sequences were aligned (Figure 2). However, when the EOLA1 structure was superimposed onto that of *Zm*ASCH, we observed that Trp27 and Trp48 of EOLA1 are similarly located to Trp15 and Tyr90 of *Zm*ASCH near the entrance of the core cavity. Accordingly, Trp27 and Trp48 in EOLA1 would form stacking interactions with a nucleotide base adjacent to the core cavity-bound nucleotide (Figure 5b). Collectively, the EOLA1 structure strongly implies its possibility to interact with nucleic acid ligands.

### 2.5. Molecules in the Core Cavity of EOLA1 Could Be Reminiscent of Nucleotide Binding

Previous studies on *Zm*ASCH revealed that the presence of divalent metal ions is essential for substrate binding and nuclease activity of *Zm*ASCH. However, a binding site for these metal ions has not been identified yet [14]. In our EOLA1 structure, we could observe strong electron densities in the core cavity, which were modeled as two sodium ions and one glycerol found in the crystallization solution and cryoprotectant solution, respectively. The sodium ions are coordinated between two hydroxyl groups of the glycerol and residues Lys21, Glu24, and Thr25 in the ‘**G**x**K**xx**E**x**R**’ motif (Figure 5c). These sodium ions are possibly coordinated at the magnesium-binding site of EOLA1 while the complexed glycerol molecule could be mimicking a nucleotide fragment. It would be reminiscent of nucleotide binding in EOLA1, in which the conserved ‘**G**x**K**xx**E**x**R**’ motif could interact with metal ions coordinating a nucleotide.

## 3. Discussion

EOLA1 was reported to mediate the expression of several proteins such as IL-6 and VCAM-1 under the stimulus of LPS [10,25]. Previously, a fraction of ASCH proteins were speculated to conduct transcriptional or translational roles [11], and the nuclease activity of *Zm*ASCH was suggested to be involved in the removal of cellular RNAs, and thus, regulation of transcriptional and translational processes [14]. In the *Zm*ASCH study, Tyr47 of *Zm*ASCH, which is conserved in EVE proteins, was observed to be crucial for nucleolytic activity [20] (Figure 2). This implies that EOLA1 would lack the nucleolytic activity of *Zm*ASCH and only be able to bind to nucleotides. The regulatory activity of EOLA1 on the expression of IL-6 and VCAM-1 could be mediated by its binding to RNA molecules and related RNA-processing machineries, similar to how the methylated adenine recognition of human YTH is involved in mRNA-splicing processes; YTH binds to pre-mRNA splicing factor SRSF3 and SRSF10 [26]. The localization of EOLA1 in both the nucleus and cytoplasm also supports its putative role as a regulator of RNA processing [25]. EOLA1 has not been listed in the RNA-interacting protein library yet, and our preliminary binding screening assay with an RNA aptamer library also did not reveal any hits (data not shown). To understand the exact biological functions of EOLA1, further studies identifying its binding partners, such as nucleic acids, proteins, or any possible cofactors, would be needed.

## 4. Materials and Methods

### 4.1. Cloning, Protein Expression, and Purification of EOLA1

A gene of full-length EOLA1 (residues 1–158) was amplified using polymerase chain reaction (PCR) and cloned into pET-28a(+) vector (Novagen, Darmstadt, Germany) between Nde1 and Xho1 sites to have a C-terminal hexahistidine-tag. The recombinant plasmid was transformed into Rosetta™ 2(DE3) pLysS (Novagen, Darmstadt, Germany), an *Escherichia coli* strain. The transformed cells were grown in Luria–Bertani media containing 100 μg/mL kanamycin at 37 °C until OD_600_ reached 0.5 and overexpression of EOLA1 was induced by addition of 0.5 mM isopropyl β-D-1-thiogalactopyranoside. The cells were incubated for additional 6 h and harvested (5.5 g). Then the cells were lysed using an ultrasonic processor (Vibra-Cell™ VCX750; Sonics, Newtown, CT, USA) in 50 ml of a buffer containing 500 mM NaCl, 20 mM Tris-HCl (pH 7.5), 35 mM imidazole, and 1 mM phenylmethylsulfonyl fluoride and centrifuged at 35,000× *g* for 60 min. The resultant supernatant was filtered with a filter device (0.45 μm; Sartorius, Göttingen, Germany) and loaded onto a 5-ml HiTrap™ Chelating HP column (GE Healthcare, Chicago, IL, USA) for affinity chromatography. The bound proteins were eluted with addition of a buffer containing 500 mM NaCl, 20 mM Tris-HCl (pH 7.5), and 1 M imidazole with an increasing gradient. The fractions containing EOLA1 were loaded onto a HiPrep™ 26/10 Desalting column (GE Healthcare, Chicago, IL, USA) and eluted with a buffer containing 20 mM Tris-HCl (pH 7.5). The desalted samples were further loaded onto a 5-ml HiTrap™ Q HP column (GE Healthcare, Chicago, IL, USA) for anion-exchange chromatography. The bound proteins were eluted with the addition of a buffer containing 1 M NaCl and 20 mM Tris-HCl (pH 7.5) with increasing gradient. Finally, the fractions containing EOLA1 were loaded onto a HiLoad™ 16/600 Superdex 75 pg column (GE Healthcare, Chicago, IL, USA) previously equilibrated with a buffer containing 200 mM NaCl and 10 mM Tris-HCl (pH 7.5) and eluted.

For the incorporation of selenomethionine (SeMet), EOLA1 was overexpressed in B834(DE3) (Novagen, Darmstadt, Germany), a methionine-auxotrophic *E*. *coli* strain. The cells were cultured in media containing M9 minimal salts (Sigma-Aldrich, Darmstadt, Germany), amino acid mix containing L-selenomethionine, and 100 μg/mL kanamycin. The overexpression and purification of SeMet-derived EOLA1 were implemented as for native EOLA1.

### 4.2. Crystallization, Data Collection, and Structure Determination

Purified proteins concentrated to 10 mg/mL were crystallized using the sitting drop vapor diffusion method at 22 °C by mixing 0.5 μL of the protein and 0.5 μL of crystallization solutions from commercial screening kits. EOLA1 crystals were grown after two days in a solution containing 4.3 M NaCl and 0.1 M HEPES-HCl (pH 7.5) (Crystal Screen 2™; Hampton Research, Aliso Viejo, CA, USA). The crystallization condition could be further optimized using the hanging drop diffusion method by mixing 1 μL of the protein (15 mg/mL) and 0.5 μL of the crystallization solution at 22 °C. Prior to x-ray diffraction experiments, crystals were cryoprotected with the crystallization solution supplemented with 10% glycerol and flash-cooled in a nitrogen gas stream of 100 K. Diffraction data were collected at the beamline PLS-5C and -7A of the Pohang Accelerator Laboratory (Pohang, Republic of Korea). The collected data were processed and scaled using the *HKL2000* program suite [27]. The model of EOLA1 was initially obtained using the SAD method with a SeMet-derived crystal using the *Autosol* program [28,29]. The final model of EOLA1, obtained from the native diffraction data using the molecular replacement method with the *Phaser* program [30], was iteratively refined with the *Coot* [31], *PHENIX.refine* [32], and *Refmac* programs [33] and validated with the *MolProbity* program [34].

### 4.3. Data Availability

The coordinates and structure factors of EOLA1 have been deposited in the Protein Data Bank (PDB) with the accession ID 5Y7D.

## Figures and Tables

**Table 1:** Statistics for data collection and model refinement.

	Endothelial-Overexpressed Lipopolysaccharide-Associated Factor 1 (EOLA1, PDB ID: 5Y7D)	SeMet-EOLA1 (SAD, Peak)
**Data collection**		
Beamline	PLS-7A	PLS-5C
Space group	*P*4_1_2_1_2	*P*4_1_2_1_2
Cell dimensions		
a, b, c (Å)	49.77, 49.77, 175.71	49.65, 49.65, 176.08
α, β, γ (°)	90, 90, 90	90, 90, 90
Wavelength (Å)	1.0000	0.9795
Resolution (Å)	50.00–1.71 (1.74–1.71) ^1^	50.00–1.93 (1.96–1.93) ^1^
No. of reflections	24953	17540
*R*_merge_^2^	0.062 (0.579) ^1^	0.081 (0.577) ^1^
<*I*>/<σ(*I*)>	53.11 (6.13) ^1^	30.28 (5.46) ^1^
Completeness (%)	99.8 (99.8) ^1^	99.9 (100.0) ^1^
Redundancy	29.4 (24.1) ^1^	15.1 (15.5) ^1^
**Refinement**		
Resolution (Å)	32.93–1.71	
*R*_work_/*R*_free_^3^ (%)	19.9/24.7	
No. of atoms	1530	
Macromolecule	1283	
Ligand/ion ^4^	26	
Water	221	
RMSD		
Bond lengths (Å)	0.005	
Bond angles (°)	1.08	
Overall B factor	31.4	
Macromolecule	29.7	
Ligand/ion ^4^	39.2	
Water	40.2	
Ramachandran favored/outliers (%)	98.1/0.0	
Poor rotamers (%)	0.0	

^1^ Values in parentheses refer to the highest resolution shell. ^2^ *R*_merge_ = Σ_h_Σ_i_ | *I*(*h*)_i_ − <*I*(*h*)> |/Σ_h_Σ_i_ *I*(*h*)_i_, where *I*(*h*) is the intensity of reflection *h*, Σ_h_ is the sum over all reflections, and Σ_i_ is the sum over i measurements of reflection *h*. ^3^ *R* = Σ| |*F*_obs_| − |*F*_calc_| |/Σ|*F*_obs_|, where *R*_free_ is calculated for a randomly chosen 5% of reflections, which were not used for structure refinement and *R*_work_ is calculated for the remaining reflections. ^4^ Two glycerols, five sodium ions, and nine chloride ions are included.

**Table 2:** Structurally similar proteins to EOLA1 based on the Dali analyses.

PDB ID	Z-score (DALI)	Cα RMSD	Description	Source
2E50	12.4	3.4	Activating signal cointegrator-1	*Homo sapiens*
5GUQ	10.4	2.3	ASCH protein	*Zymomonas mobilis*
2DP9	9.2	2.4	TTHA0113	*Thermus thermophilus*
2KKU	7.1	3.0	Uncharacterized protein AF_2351	*Archaeoglobus fulgidus*
2EVE	6.2	3.7	EVE protein	*Pseudomonas syringae*
